# Altered postural timing and abnormally low use of proprioception in lumbar spinal stenosis pre- and post- surgical decompression

**DOI:** 10.1186/s12891-019-2497-0

**Published:** 2019-05-01

**Authors:** Sarah Kneis, Verena Bruetsch, Daniela Dalin, Ulrich Hubbe, Christoph Maurer

**Affiliations:** 1 Department of Neurology, Faculty of Medicine and Medical Center – University of Freiburg, Freiburg, Germany; 2 Department of Neurosurgery, Faculty of Medicine and Medical Center – University of Freiburg, Freiburg, Germany

**Keywords:** Low back pain, Posturography, Postural balance, Center of pressure, Walking speed, Quality of life

## Abstract

**Background:**

Lumbar spinal stenosis (LSS) is frequently associated with postural instability. Although several studies evaluated patients’ functional impairments, underlying sensorimotor mechanisms are still poorly understood. We aimed to assess the specific set of postural control deficits associated with LSS during spontaneous and externally perturbed stance and evaluated post-surgical changes in postural behavior.

**Methods:**

We analyzed postural control in eleven LSS patients (age 69 ± 8 years) pre- and post-laminectomy, correlated experimental data with functional tests and patient-reported outcomes, and compared findings to 15 matched, healthy control subjects (age 70 ± 6 years). Postural control was characterized by spontaneous sway measures and measures of perturbed stance. Perturbations were induced by anterior-posterior pseudorandom tilts of the body support surface. We used an established postural control model to extract specific postural control parameters.

**Results:**

Spontaneous sway amplitude, velocity and frequency were abnormally large in LSS patients. Furthermore, patients’ postural reactions to platform tilts, represented by GAIN and PHASE were significantly altered. Based on simple feedback model simulations, we found that patients rely less on proprioceptive cues for stance regulation than healthy subjects. Moreover, their postural reactions’ timing is altered. After surgery, patients’ spontaneous sway amplitude was significantly reduced and their postural timing approximated the behavior of healthy subjects.

**Conclusion:**

The reduction in proprioceptive input for stance control due to stenosis-caused afferent dysfunction is a functional disadvantage for LSS patients – and may be the basis of increased spontaneous sway. This disadvantage may cause the timing of postural reactions to alter, with the intent of preventing rapid changes in stance regulation for safety reasons. After surgery, patients’ postural timing approximated those of healthy subjects, while the abnormally low use of proprioception remained unchanged. We suggest the post-surgery rehabilitation of proprioception, eg through balance exercises on unstable surfaces and reduced visual input.

## Background

Degenerative lumbar spinal stenosis (LSS), which is commonly diagnosed between the 6th and 7th decade [1, 2], is often accompanied by functional impairments like gait and balance problems [3–9]. It is a frequent cause for spinal claudication and can lead to chronic or recurrent low back pain (LBP) syndrome [1, 2], which, in turn, is increasingly prevalent in industrial nations [10]. LSS can substantially impair i.a. patients’ daily living, social participation, and physical function [11, 12]. Therapeutic approaches include pain reduction ranging from exercise- and physiotherapy to the short-term administration of analgesics and muscle relaxants [13, 14]. If those approaches fail, surgical intervention is an option [13, 14].

Many LSS patients suffer from considerable functional impairments that increase their risk of falling [7, 8] and subsequent adverse health events. Understanding the functional deficit of LSS patients may facilitate the planning of suitable therapy strategies. While studies of functional deficits explicitly in LSS patients [4, 5, 7, 15, 16] are seldom, the balance performance of patients with LBP (presumably including LSS patients without specific nerve root irritations) has been more frequently assessed see [17]: several studies reported modified postural control in LBP patients compared to healthy control subjects [18–28]. Evaluating body excursion especially under dynamic and/or limited visual conditions revealed larger postural sway, represented by the root mean square (RMS) [17, 18, 21, 23, 29] and center of pressure (COP) displacement [19, 22, 25, 29]. Furthermore, the mean velocity (MV) of COP increases significantly in LBP patients when closing their eyes [17]. However, causes for LBP are manifold and may trigger different underlying mechanisms leading to balance impairments, such as pain, or afferent impairments. There is little knowledge about postural control behavior in the various subgroups of LBP patients.

Studies explicitly targeting LSS usually rely on COP evaluations during quiet stance [4, 5, 7, 15], while underlying sensorimotor mechanisms of stance regulation in LSS patients have been seldom assessed. Initial indications about altered body sway in LSS patients were published by Hanai et al. [15], who found that COP displacements after the onset of claudication increased and moved forward, in line with Sasaki et al. [7]. After surgery, patients’ body sway decreased and approximated those of healthy subjects [15]. Other studies [4, 5, 7] supported the observation of abnormally large COP displacements in LSS patients, especially in anterior-posterior direction. Iversen et al. [4] found, in addition, significantly reduced functional-reach performance that may indicate impaired dynamic balance and an increased risk of falling. They suspect that LSS patients sense only greater body excursions in terms of a proprioceptive impairment, and need longer to switch between an assumed “open-loop”-control on the spinal level and an assumed “closed-loop” control on the cerebral level [4]. Both Iversen et al. [4] and Leinonen et al. [30] observed impaired lumbar movement perception that may result from a peripheral sensory loss. Furthermore, they propose an association between postural instability and abnormal motor-evoked potentials, while somatosensory conduction would not influence postural stability [30].

Based on those studies, we hypothesize that LSS patients reveal abnormal postural control that may be due to a reduced use of afferent, proprioceptive information. Instead, LSS patients may increase their dependence on space cues for postural stability. The present study was undertaken to i) assess the specific set of postural control deficits associated with LSS during spontaneous and externally perturbed stance and ii) to evaluate post-surgical changes in postural behavior. We applied an established postural control model [31–33] to extract relevant postural-control parameters that may reveal underlying sensorimotor mechanisms responsible for LSS patients’ balance problems.

## Methods

### Patients

During 19 weeks, we consecutively enrolled 11 LSS patients and 15 healthy control subjects. Since our study follows a basic approach with the aim to understand fundamental postural control differences between LSS patients and healthy subjects, sample size could not be based on a previous power calculation. Inclusion criteria were: LSS diagnosis; hospitalized at Neurocenter – University of Freiburg, Germany and scheduled for surgical decompression. The lumbar spinal stenosis in our patients was due to an acquired degeneration and consecutive narrowing of the central canal leading to neurogenic claudication. We excluded patients suffering from radiculopathy. Other exclusion criteria were: additional disorders of the musculoskeletal system like lumbar disc herniation, synovial cyst, bone metastasis, lateral recess stenosis, recessusstenose, symptomatic cervical stenosis, paresis of knee extensors and hip flexors, or other sensorimotor impairments like polyneuropathy, eg due to diabetes mellitus, M. Parkinson, Myasthenia gravis, symptomatic stroke, clonus, cerebellar disorders, paraplegia, tumors or bleedings, peripheral vascular diseases like peripheral arterial occlusive disease; frailty, sarcopenia, > 80 years. Therefore, all patients underwent detailed clinical neurological examination by an experienced neurologist. Control subjects were matched according to sex, age, weight and height. Table 1 summarizes our subjects’ characteristic.

**Table 1:** Patients’ characteristic with *p*-values for differences

	Patients *N* = 11	Patients post-surgery *N* = 9	Control subjects *N* = 15	*P*
Age mean ± SD	69 ± 8		70 ± 6	0.89
Sex (m:f) *N*	7: 4		8: 12	0.71
Weight (kg) mean ± SD	84.3 ± 17.9		77.9 ± 14.1	0.08
Height (m) mean ± SD	1.71 ± 0.7		1.69 ± 1.0	0.22
BMI (kg/m^2^) mean ± SD	28.9 ± 5.3		27.2 ± 3.6	0.14
Location of LSS *N*				
- L 2/3 and L 3/4	1			
- L 2/3, L 3/4 and L 4/5	2			
- L 3/4	3			
- L 3/4 and L 4/5	2			
- L 3/4, L 4/5 and L 5 / S 1	1			
- L 4/5	2			
Time between symptom onset and surgery (years) mean ± SD	2.5 ± 1.6			
Time between diagnosis and surgery (months) mean ± SD	6.1 ± 3.9			
Symptoms *N*				
- Claudicatio spinalis	11			
- Reduced walking distance	11			
- Subjective leg muscle weakness	11			
- Inflected trunk position during walking	11			
- Pain reduction induced by trunk flection and sitting position	11			
Surgery *N*				
- Minimally invasive osteoligamentous decompression	7			
- Partially hemilaminectomy with additional osseous decompression	4			
Days between pre- and post-assessment mean ± SD		59 ± 13		
Physiotherapy between surgery and post-assessment *N*		9		

*P* p-value, *SD* standard deviation, *BMI* body mass index, *LSS* lumbar spinal stenosis

Patient-reported outcomes and postural control assessments were assessed pre-surgery (pre) and six to twelve weeks post-surgery (post) at routinely post-surgery check-up. During this interval, 9 patients received routine physiotherapy, with a focus on balance training as a standard measure for LSS patients after surgical decompression. All patient-related assessments were conducted by the same examiner following standardized measures.

This study was approved by the Ethics Commission of University Medical Center Freiburg. All subjects provided written informed consent to the experimental procedure in accordance with the Declaration of Helsinki.

### Functional tests and patient-reported outcomes (PROs)

We clinically tested balance and mobility via the Performance Oriented Mobility Assessment (POMA) [34], Berg-Balance Scale (BBS) [35], the Clinical Test for Sensory Interaction in Balance (CTSIB) [36], the Functional Reach- [37], the Timed Up and Go (TUG)-, Chair Rising (CRT)- and 10 m walking test [38] as well as spine mobility by determining the distance between the fingertips and ground.

Moreover, patients were asked to describe their pain intensity during resting and stress via visual analogue scale, and their maximum walking distance until claudicatio spinalis. Health-related quality of life was assessed via the SF-36 questionnaire (the short form (36) health survey) [39] and self-reported functional impairments via the Hannover questionnaire for functional disability caused by back pain (FFb-H-R) [40].

### Procedure

Postural control assessments, ie *spontaneous sway* and *perturbed stance* were measured with a custom-built motion platform under two *visual conditions*, with eyes open and with eyes closed [41, 42]. Each trial lasted one minute. The participants were told to stand upright on the platform in comfortable shoes. Stance width was predetermined within a marked area. For safety reasons, participants had to hold two ropes hanging from the ceiling in crossed-arms position so that they could not perceive a somatosensory spatial orientation signal (for a more detailed setup description, see 32, [43]).

*Spontaneous sway* was measured on the non-moving platform. The COP sway path was detected with a force-transducing platform (Kistler platform type 9286, Winterthur, Switzerland).

*Perturbed stance* was measured on a moving platform to differentiate sensory contributions in reaction to external disturbances. We analyzed rotational tilts in the sagittal plane with the tilt axis passing through the participant’s ankle joints. As the stimulus profile followed a pseudorandom stimuli (PRTS, pseudorandom ternary sequence [31]) with two peak angular displacements (*stimulus amplitude*: 0.5° and 1° peak-to-peak) and analyzed at eleven *stimulus frequencies* (0.05, 0.15, 0.3, 0.4, 0.55, 0.7, 0.9, 1.1, 1.35, 1.75 and 2.2 Hz).

Angular and translational excursions of the lower (hip-ankle: hip movement) and upper (shoulder-hip: shoulder movement) *body segments* and of the platform in space were measured using an optoelectronic motion-measuring device (Optotrak 3020, Waterloo, Canada) with markers attached to shoulder, hip, and the platform. Each marker consisted of three light-emitting diodes fixed to a rigid triangle. The triangles were fixed on the participant’s hips and shoulders and to a rigid bar on the platform. 3-D angular and translational positions of the triangles were used to calculate marker positions. Optotrak® and Kistler® output signals as well as the stimulus signals were transferred on-line to a computer system (IBM compatible Pentium®) via an analogue-digital converter at a sampling rate of 100 Hz. We recorded all data with software programmed in LabView® (National Instruments, Austin, Texas, USA).

### Model simulations

The model simulations were implemented in Simulink/ MATLAB™, as was an optimization procedure we used to identify model parameters [31, 33, 43]. In brief, the procedure varied defined model parameters, using the Matlab Optimization toolbox function “fminsearch” (which is based on the simplex search method of Nelder-Mead) in order to minimize the deviation between the simulated responses and corresponding experimental data. With each search’s iteration, simulated responses to the pseudorandom stimulus cycles were obtained, the simulated data were analyzed in the same manner as the experimental data, and a scalar error function was evaluated representing the difference between simulated and experimental results. Then, the search procedure changed the parameters and the error function was re-evaluated. This sequence was repeated until parameters were identified yielding a minimum of the error function.

The model exploited here includes a negative feedback loop that relates body excursion detected by visual, vestibular, and proprioceptive sensors to a corrective torque via a neural controller (see [43]). We estimated the neural controller’s parameters with proportional (*Kp*), derivative (*Kd*) and integral (*Ki*) contributions (PDI-controller). Neural controller gains are, in part, determined by mass and height of each subject’s center of mass (*COM*) (see [31]).

Moreover, we derived time delay (*Td*), proprioceptive sensory weight (*Wp*), and biomechanical elasticity (*Ppas*) and damping (*Dpas*) of the muscles and tendons. We fitted model simulations to experimental transfer functions under different stimulus amplitudes and visual conditions.

### Data analysis

Data was analyzed off-line with custom-made software programmed in MATLAB® (The MathWorks Inc., Natick, MA, USA). From the COP, lower (hip) and upper (shoulder) body excursions over time in anterior-posterior and medio-lateral *sway directions,* we calculated *RMS* around the mean COP position. After differentiating the time series, we calculated *MV*. In addition, center frequency (CF) was extracted from the power spectrum (see also [44, 45]). Transfer functions from stimulus-response data were calculated via a discrete Fourier transform. Fourier coefficients of stimulus and response time series are used to determine *GAIN* and *PHASE* of the responses with respect to stimulus frequencies. We used these functions as the experimental data basis for model simulations using an upright-stance model. Further analysis was done using Microsoft Excel and statistic programs (JMP®, SAS Institute Inc., Cary, NC, USA). Statistical significance was tested by multivariate analysis of variance. The level of statistical significance was set at *p* = 0.05. Relationships between the functional tests, RPOs and our postural control parameters obtained from platform measures were analyzed via the Pearson correlation test.

## Results

### Spontaneous sway

**Fig. 1:**
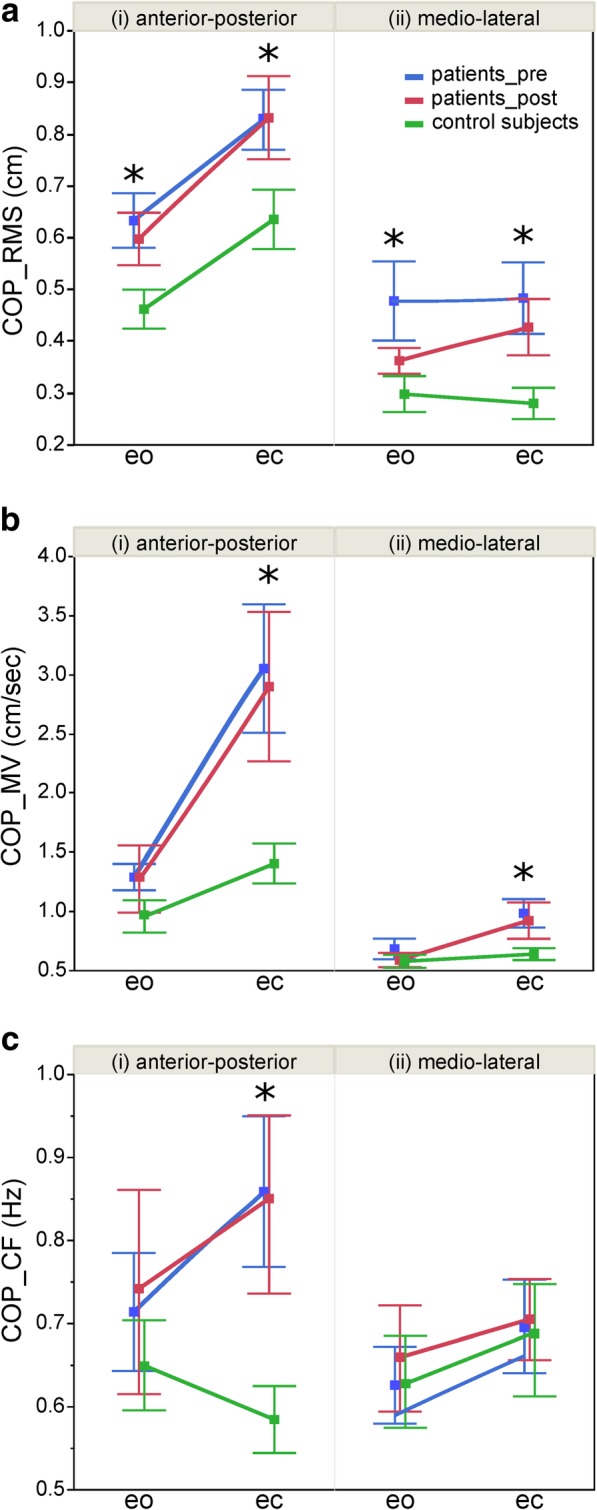
Spontaneous sway. Center of pressure (COP) parameters: mean and standard error of (**a**) root mean square (RMS), (**b**) mean velocity (MV) and (**c**) center frequency (CF) of COP sway in (i) anterior-posterior and (ii) medio-lateral direction each for eyes open (eo) and eyes closed (ec) condition in healthy control subjects, patients before (pre) and after (post) surgery. Significant differences between healthy control subjects and patients before (pre) surgery are indicated with an asterisk

Our patients’ postural sway was greater than our control subjects’ (RMS: F = 34.91; p = < 0.0001; see Table 2). Post-surgery, RMS was significantly reduced (F = 4.89; *p* = 0.0289; see Table 2). This finding was mainly based on the eyes-open condition (interaction F = 9.32; *p* = 0.0028) and in medio-lateral direction (interaction F = 5.27; *p* = 0.0234, Fig. 1a).

**Table 2:** Posturography

	Patients *N* = 11	Patients post-surgery *N* = 9	Control subjects *N* = 15	*P*
	Mean (SE)	Δ (%)	Mean (SE)	
Spontaneous sway				
RMS (cm)	0.56 (0.02)		0.43 (0.01)	< 0.000*
		−5.3		0.029*
MV (cm/sec)	0.72 (0.04)		0.52 (0.03)	0.002*
		−6.4		0.244
CF (Hz)	0.50 (0.50)		0.44 (0.01)	0.001*
		4.6		0.071
Perturbed stance				
Mean GAIN	1.85 (0.02)		1.93 (0.02)	0.015*
		1.2		0.482
Mean PHASE (°)	−98.83 (2.22)		−119.09 (1.90)	< 0.000*
		15.3		< 0.000*
Model parameters				
Ki (s ^−1^·rad^− 1^)	74.95 (2.63)		74.85 (2.25)	0.979
		5.2		0.407
Kp (rad^−1^)	1093.64 (39.11)		1047.63 (33.49)	0.374
		0.0		0.994
Kd (s·rad^−1^)	307.39 (8.61)		285.62 (7.37)	0.058
		−4.3		0.206
Wp (%)	61.04 (2.59)		68.81 (2.22)	0.025*
		3.1		0.595
Td (msec)	172 (6.1)		172 (5.3)	0.919
		−5.0		0.344
Ppas (rad^−1^)	82.25 (3.37)		82.88 (2.88)	0.888
		−9.0		0.490
Dpas (s·rad^−1^)	55.93 (1.42)		57.09 (1.21)	0.533
		6.3		0.296

*indicates a significant difference *p* < 0.05

*Δ* post-surgery minus pre-surgery, *SE* standard error, *RMS* root means square, *MV* mean velocity, *CF* center frequency, *Ki* integral gain of the Neural Controller, *Kp* proportional gain (stiffness factor), *Kd* derivative gain (damping factor), *Wp* proprioceptive sensory weight, *Td* feedback time delay, *Ppas* passive stiffness factor, *Dpas* passive damping factor

Patients exhibited a higher MV at all conditions and qualities than control subjects (F = 9.82; *p* = 0.0019; see Table 2). Group designation significantly interacted with visual condition (F = 8.28; p = < 0.0042) and sway direction (F = 7.84; p = < 0.0054). Differences between patients and control subjects were especially pronounced with eyes closed and in the anterior-posterior direction (Fig. 1b). Post-surgery, we detected no effect on MV.

Patients swayed at higher frequencies than healthy subjects (CF: F = 10.58; *p* = 0.0013; see Table 2), descriptively due to differences in anterior-posterior direction and with eyes closed (no significant interaction; see Fig. 1c). Post-surgery, we observed no significant changes in CF.

### Perturbed stance

The transfer function between platform tilt and body angular displacement is characterized by GAIN and PHASE behavior.

**Fig. 2:**
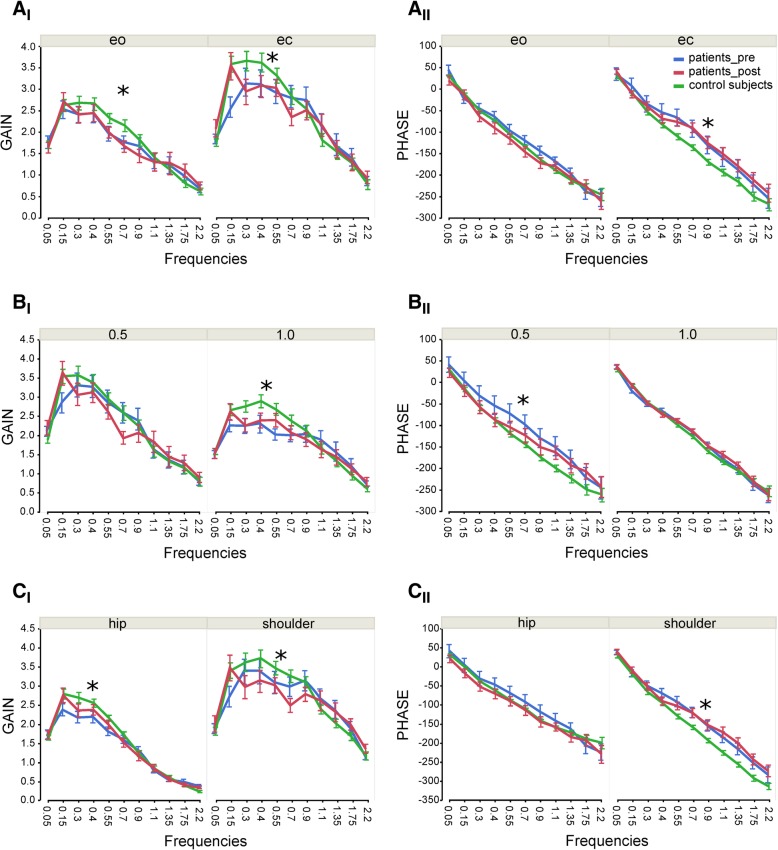
Transfer functions. Mean and standard error of GAIN (**A**_I_ – **C**_I_) and PHASE (**A**_II_ – **C**_II_) behavior as a function of frequency for eyes-open (eo) and eyes-closed (ec) condition (**A**), for 0.5° and 1° rotational amplitude (**B**) and body segments (**C**) in healthy control subjects, patients before (pre) and after (post) surgery. Significant differences between healthy control subjects and patients before (pre) surgery are indicated with an asterisk

Patients’ GAIN was significantly smaller than that of control subjects (F = 5.97; *p* < 0.0146; see Table 2). In addition, group designation interacted significantly with frequency (F = 3.53; *p* = 0.0001): at very low and high frequencies, patients showed a slightly larger GAIN than control subjects, while at lower and mid-range frequencies patients’ GAIN was smaller than control subjects’ (Fig. 2A_I_ – C_I_). We observed no interaction between GAIN and visual condition or rotational amplitude or body segment. Post-surgery, we observed no changes in GAIN behavior.

Patients displayed less PHASE lag than control subjects (F = 48.29; *p* < 0.0001; see Table 2). Moreover, group designation interacted significantly with rotational amplitude (F = 45.32; p = < 0.0001; Fig. 2B_II_). In particular, patients’ PHASE behavior varied between the amplitudes meaning a smaller PHASE lag at 0.5° compared to 1°, while control subjects acted similarly across amplitudes leading to a group difference at 0.5° amplitude (Fig. 2B_II_). Patients demonstrated a greater PHASE lag post-surgery than pre-surgery (F = 14.12; *p* = 0.0002; see Table 2). Patients’ post-surgery PHASE behavior interacted with rotational amplitude (F = 17.7; p < 0.0001): PHASE advance was more pronounced at 0.5° than at 1°; thus patients’ post-surgery PHASE behavior approximated our control subjects’ values at lower and mid-range frequencies, while the patients’ (pre- and post-surgery) and control subjects’ PHASE behavior at 1° were nearly identical (Fig. 2B_II_). Furthermore, patients’ post-surgery PHASE behavior interacted with body segment (F = 5.5; *p* = 0.0042): patients’ PHASE lag post-surgery at hip increased at lower and mid-range frequencies approximating the range of control subjects (Fig. 2C_II_).

The following results are derived from the model-based approach described above, and present the relevant parameter differences between patients and control subjects.

We observed no significant effects for the integral (Ki), proportional (Kp), and derivative (Kd) parts of the neural controller (Table 2). However, the proportional (Kp) and derivative (Kd) part were slightly larger in patients than in control subjects, whereas the Kd difference almost reached the significance level (0.058).

Time delay between stimulus and response (Td), passive muscle stiffness and damping (Ppas and Dpas) did not differ significantly between patients and control subjects (Table 2).

**Fig. 3:**
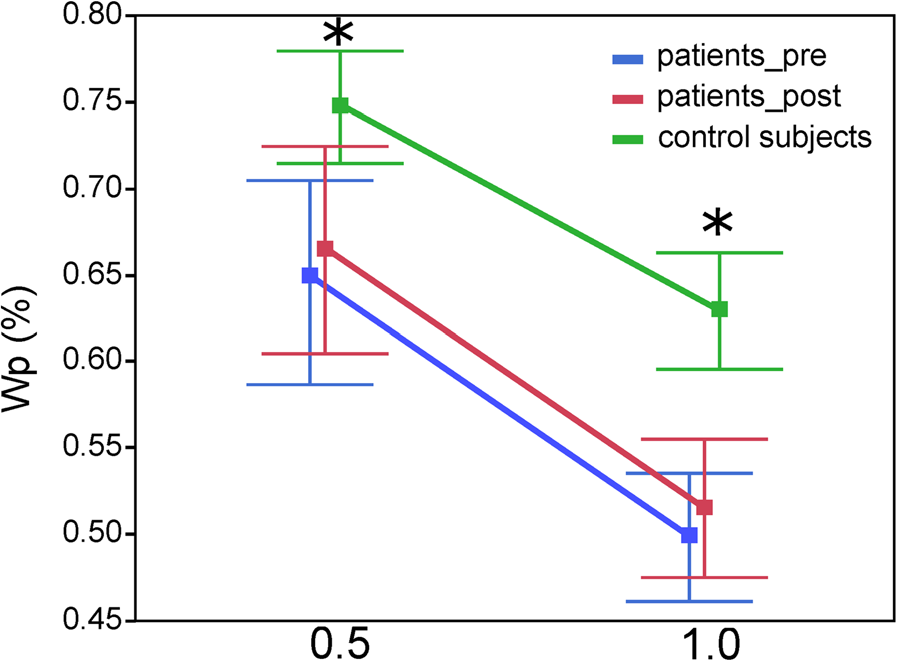
Model parameters. Mean and standard error of the proprioceptive sensory weight (Wp) for 0.5° and 1° rotational amplitude in healthy control subjects, patients before (pre) and after (post) surgery. Significant differences between healthy control subjects and patients before (pre) surgery are indicated with an asterisk

Only, the sensory weighting factor Wp, indicating the proportion of proprioceptive vs. vestibular and visual cues, differed significantly between patients and control subjects (F = 5.21; *p* = 0.0246; Fig. 3). Whereas patients rely on average 61% on proprioceptive cues and hence 39% on spatial cues, control subjects rely 69% on proprioceptive and 31% on spatial cues (see Table 2). This Wp effect did not interact with visual condition or rotational amplitude, and was un affected by surgery, as with any other model parameters.

### Functional tests and PROs

Patients’ pre-surgical functional status was not considerably impaired (Table 3). After surgery, we detected no significant change in functional tests. However, among PROs, patients reported impaired function (FFb-H-R), reduced quality of life (SF-36), limited maximum walking distance and high pain intensity during stress (Table 3). After surgery, pain intensity during rest (F = 5.73; *p* = 0.0436) and stress (F = 43.75; *p* = 0.0002) was significantly reduced; patients reported a considerably longer walking distance (F = 7.44; *p* = 0.0295) and improved function (F = 8.76; *p* = 0.0182), while quality of life was unaffected.

**Table 3:** Functional tests and patient-reported outcomes (PROs)

	Patients *N* = 9	Patients post-surgery *N* = 9	Mean difference (95%CI)	*P*
	Mean	Mean		
Functional tests				
Spine mobility (cm)^a^	9.89	10.00	0.11 (4.13 − −3.91)	0.951
Functional reach (cm)^b^	32.05	34.11	2.06 (11.95 − − 7.84)	0.645
BBS (scale max. 56)^c^	52.67	54.33	1.67 (4.27 − − 0.94)	0.179
10 m walking test (sec)	6.8	5.7	−1.1 (0.3 − − 2.4)	0.099
CRT (sec)^d^	16	18	2 (5 − − 1)	0.250
CTSIB (scale 6–24)^e^	9.3	9.6	0.2 (1.4 − − 0.9)	0.665
POMA (scale max. 28)^f^	26.7	27.3	0.67 (1.5 − −0.2)	0.111
TUG (sec)^g^	10.0	8.9	− 1.1 (0.5 − − 2.7)	0.154
PROs				
Pain intensity during rest (VAS scale 0–10)	2.11	0.67	−1.44 (−0.05 − − 2.84)	0.044*
Pain intensity during stress (VAS scale 0–10)	7.56	3.67	− 3.89 (− 2.53 − − 5.35)	< 0.000*
Max. walking distance (m)	329	4775	4446 (8302–591)	0.030*
FFb-H-R (score max. 100%)^h^	60.7	73.2	− 12.5 (− 2.8 − − 22.2)	0.018*
SF-36 (score 0–100%)^i^	49.3	56.4	7.2 (23.0 − −8.6)	0.326

*indicates a significant difference *p* < 0.05

*P p*-value, *CI* confidence interval, *BBS* Berg Balance Scale, *CRT* Chair Rising Test, *CTSIB* Clinical Test for Sensory Interaction in Balance, *POMA* Performance Oriented Mobility Assessment, *TUG* Times Up and Go test, *VAS* Visual Analogue Scale, *FFb-H-R* Hannover questionnaire for functional disability caused by back pain

^a^Spine mobility is determined by the distance between the fingertips and ground in cm

^b^< 25 cm indicates an increased fall risk

^c^< 54 indicates an increased fall risk

^d^Duration in sec for five sit-to-stand repetitions

^e^A higher score indicate greater balance problems

^f^< 24 indicates an increased fall risk

^g^<10s indicates unlimited mobility

^h^> 100%–80% normal function. 70%–80% moderate impaired function. 60% − 70 impaired function. < 60% clinical-relevant functional disability

^i^A higher score represents less disability

### Correlations

Our correlation analysis revealed a strong interrelation between the functional tests BBS, 10 m walking test, POMA and TUG (r > 0.7, *p* < 0.001), as well as between CTSIB and POMA (r = 0.59, *p* < 0.01), while CRT correlated only with the 10 m walking test (r = 0.54, p = 0.02) and TUG (r = 0.65, p < 0.01). In contrast, functional reach and spine mobility did not correlate with other functional tests or with PROs or with experimental results. Based on the largely strong correlations between BBS, 10 m walking test, POMA, TUG, CRT, and CTSIB we used a principal component (PC) analysis to condense these functional tests (FT) into one variable (PC-FT).

Concerning PROs, we noted significant correlations between FFb-H-R, which represents the center of PROs’ correlations and reflects many different aspects of patient-reported functional impairments, and pain intensity during stress as well as max. Walking distance (r > 0.5, *p* < 0.05). Moreover, FFb-H-R correlated strongly with SF-36 (r = 0.63, *p* = 0.005). Walking distance correlated strongly with pain intensity during stress (r = 0.79, p < 0.001) and with SF-36 (r = 0.49, *p* = 0.05).

Pain intensity during rest showed no interrelation to other PROs, any functional tests, or experimental results.

Furthermore, we observed a strong correlation between experimental results, ie PHASE and GAIN (r = 0.59, *p* = 0.009), while the model parameter representing proprioception, i.e. Wp, correlated only with GAIN (r = 0.71, *p* = 0.001). GAIN and PC-FT also correlated strongly (r = 0.62, *p* = 0.006), as did GAIN and FFb-H-R (*p* = 0.52, *p* = 0.027). FFb-H-R correlated also with PHASE (r = 0.50, *p* = 0.035) and with PC-FT (r = 0.67, *p* = 0.003).

Spontaneous sway results did not correlate with perturbed stance results, with functional tests, or with PROs.

## Discussion

Functional impairments are common among LSS patients [3, 6, 9]. Some authors have suggested [4, 30] that these impairments may be related to peripheral sensory loss. However, it remains unclear which postural mechanisms are directly involved. Possible candidates are impaired motor actuator, sensorimotor time delay, impaired weighting of visual, vestibular and proprioceptive input, passive muscle elasticity, and damping. This ambiguity motivated us to conduct the present study. Our study’s aim was to identify the postural control parameters that may reveal underlying sensorimotor mechanisms responsible for LSS patients’ balance problems. Moreover, we aimed to monitor pre-post-surgical changes in these parameters. We hypothesized that the main effect in LSS patients concerns the altered weighting of sensory information. In particular, we expected an altered use of proprioceptive information in LSS patients as compared to healthy control subjects.

In line with earlier studies reporting postural instability in patients suffering from LSS [4, 5, 15], we detected altered postural behavior in LSS patients. Interestingly, we were able to relate these findings to a modified use of sensory information and to timing aspects of postural reactions. Surgery and post-surgery rehabilitation mainly affected these timing aspects of postural behavior. In addition, the clinical outcome of LSS patients post-surgery was improved, reflected by reduced patient-reported functional impairments.

Since this study serve as basis for further investigations aiming to understand the underlying sensorimotor mechanisms responsible for LLS patients’ postural instability, sample size was not based on a previous power calculation. The small sample size may limit our study’s results regarding stronger differences between groups. Furthermore, we propose to monitor post-surgery physiotherapy measures or to intervene specifically in order to control influencing factors on postural stability, and to expand the observation duration to detect long-term effects.

### Spontaneous sway

Spontaneous sway measurements revealed that LSS patients’ postural sway was greater, faster and at higher frequencies compared to matched healthy control subjects. Those alterations have been also described in LBP patients [18–29] and elsewhere in LSS patients [4, 5]. Abnormal spontaneous sway was associated with a higher risk of falling in LSS patients [7]. Our PROs confirmed functional limitations in LSS that considerably affect patients’ quality of life, and additionally indicate stress-dependent worsening of impairments.

Post-surgery, postural sway (as measured by RMS) was reduced and approximated the values of control subjects which paralleled findings from an earlier study [15]. This reduction may indicate that patients achieved better postural stability post-surgery following the decompression of lumbar radices, which carry the motor and sensory information for lower extremity control. Specifically, the RMS reduction after surgery and rehabilitation measures may be a consequence of improved peripheral proprioception. Since surgery significantly reduced pain intensity and patient-reported functional limitations, as expected, this also may contribute to improved stance stability, which is also influenced by rehabilitation measures.

### Perturbed stance and model-based analysis

In addition to investigating spontaneous sway, we assessed postural reactions to external stimuli. Specifically, we compared patients’ postural sway to continuous pseudorandom platform tilts in anterior-posterior direction. These raw angular traces were transformed into transfer functions in the frequency domain, reflecting patients’ reactive body excursions as a function of stance perturbation. These transfer functions revealed a smaller GAIN in patients than control subjects, especially in lower and mid-range frequencies. In other words, patients’ postural reactions were, on average, smaller than control subjects’. Furthermore, patients presented a smaller PHASE lag, representing a relatively earlier postural reaction to external stimuli than control subjects. Interestingly, PHASE differences between patients and control subjects were amplitude-dependent (apparent at 0.5°, but vanished at 1°). Patients’ PHASE lag at 0.5° corresponded to that of control subjects at 1°, meaning that patients reacted to smaller platform excursions like control subjects to greater excursions. Post-surgery, patients approximated the PHASE behavior of control subjects, which is mainly based on changes at 0.5 rotational amplitude and at hip movement.

On a first glance, LSS patients’ smaller and earlier reactive body excursions, as compared to control subjects, do not seem to be disadvantageous for postural stability. Even more surprising, post-surgery we observed a prolongation of postural reactions in LSS patients, represented by an increased PHASE lag. How could this prolongation be related to the fact that patients achieved enhanced posture stability post-surgery, as our spontaneous sway findings demonstrate (RMS)?

The transfer function findings reported above formed a basis for a model-based data interpretation: Extracting model-parameters after fitting simulated to experimental transfer functions revealed slightly more pronounced velocity control (damping) in the postural system (Kd) in patients compared to control subjects. Kd results are in line with the patients’ aforementioned PHASE behavior. Furthermore, patients use significantly less proprioceptive information, as reflected by smaller values for proprioceptive weight (Wp), than control subjects. No other model parameters differed between groups.

Following the assumption that LSS patients weigh their sensory information differently than healthy subjects, we hypothesized that patients rely relatively more on space cues and less on proprioceptive cues, as they suffer from stenosis-induced afferent dysfunction. Our GAIN results support this assumption, since our patients revealed a smaller GAIN, indicating their stronger orientation towards space coordinates and less dependency on platform movements [31, 43]. In contrast, healthy subjects integrated more proprioceptive information for stance regulation resulting in a greater GAIN. These findings are emphasized by our model-based approach, where we found patients’ proprioceptive cues (Wp) down-weighted and as a result, space cues implicitly up-weighted when compared to healthy subjects. However, up-weighting space cues implies a functional disadvantage, as the vestibular system carries a larger amount of sensory noise [46], leading to less accurate stance regulation. There is evidence that larger platform perturbations generally lead to a relative smaller GAIN, stronger orientation on space cues, a smaller PHASE lag, and greater velocity control (Kd) [47] to ensure stance stability. We assume that patients’ postural behavior might represent a compensation strategy, where patients try to prevent excessive and rapid changes in postural position already at small excursions by reducing PHASE lag due to their stenosis-caused physiological limitations and related pain. Additionally, as mentioned earlier, afferent information might be inadequate, which could be a reason why patients down-weigh proprioception. Patients compensate for their afferent dysfunction by staying within a more stable and secure range of regulation [48], while accepting disadvantages in the accuracy of postural control through increased vestibular noise. Surgery alleviated physiological limitations responsible for this compensation, leading to a PHASE-shift towards healthy subjects’ postural behavior.

The concentration of the intervention-induced PHASE effect on the hip joint may indicate, in addition, a change in hip-strategy [49, 50]. Concerning LBP patients, some authors reported that these patients exhibit a less effective hip strategy than healthy subjects [24, 27, 51]. According to those findings, we speculate that patients stiffened their hip joint pre-surgery by enhancing muscular co-contractions due to the aforementioned worries about safety. Post-surgery, this stiffness may be relaxed to enable larger body excursions [50], as surgery directly alleviates their primary impairments, eg pain [3, 9, 52].

Considering patients’ afferent dysfunction before surgery, one would assume that motor latency as reaction of the perturbation would be prolonged. However, time delay (Td) extracted from the model-based approach did not differ between groups, highlighting the assumption that altered PHASE behavior as a function of time is not primarily caused by physiological impairments but rather by a compensation strategy, ie altered timing for stance regulation.

Additionally, the model-based total effort to correct the difference between actual and desired body angle (represented by the model parameter Kp) was similar in both groups, indicating that the intensity of motor response was also not unaltered in LSS patients. We therefore assume that the stenosis-caused efferent dysfunction was less pronounced in our patients and did not lead to a fundamental deficit in relation to their stance regulation.

### Correlations

When comparing our experimental results with clinical tests of motor function and patient-reported outcomes, one is tempted to associate the spontaneous sway abnormalities, ie larger (RMS) and faster (MV) postural sway to the other functional disadvantages pre-surgery. In the literature, these spontaneous sway abnormalities are reported to be associated with postural instability and functional impairments [4, 53]. Interestingly, our spontaneous sway parameters neither correlated with patient-reported outcomes, nor with functional tests, nor with measures derived from perturbed stance. In contrast, measures from perturbed stance significantly correlate with both patient-reported functional outcomes (FFb-H-R) and specific functional tests, which in turn correlated strongly. The significant correlation between FFb-H-R and many functional tests (PC-FT: POMA [34], BBS [35], CTSIB [36], TUG-, CRT- and 10 m walking test [38]) indicate that these functional tests mainly determine the perception of functional impairments in patients [54], while other tests, ie the Functional Reach test [37], or spine mobility (distance between the fingertips and ground) seem to reflect other functionality dimensions. We claim that the close correlation between our experimental results from perturbed stance, many functional tests (PC-FT), and the patient-reported deficits may be applied in future to lower the number of tests necessary to accurately assess all facets of postural impairments.

In general, our data lead us to conclude that patients’ postural reactions following external perturbations are suitable to precisely quantify most of their postural impairments unlike spontaneous sway measures. However, taking spontaneous sway measurements is the current standard when assessing patients’ functional status [18–25, 29]. Based on our results, we suspect that parameters recorded during quiet stance fail to capture patients’ gait and stance disturbances sufficiently - thus we strongly propose applying external perturbation when assessing patients’ balance ability.

## Conclusion

In summary, LSS patients reported significant functional impairments that can be captured by our perturbed stance experiments. Analyzing perturbed stance revealed alterations in the timing of postural reactions and in the down-weighting of proprioceptive cues, possibly due to stenosis-caused afferent dysfunctions. In return, patients involuntarily accept functional disadvantages when up-weighting space cues. They may be compensating for this disadvantage by an earlier motor reaction as response to body excursions to prevent excessive and rapid changes in posture position for safety reasons. After surgery and few weeks rehabilitation, patients reported fewer impairments. Furthermore, these interventions corrected for the compensation mechanism, as patients approximated the postural behavior of healthy subjects in terms of timing aspects. However, sensory weighting was unaffected at follow-up. Thus, we propose that post-surgery rehabilitation in LSS patients should focus on interventions ameliorating the proprioceptive part of postural control, eg on balance exercises on unstable surfaces entailing reduced visual input.

## Abbreviations

BBS: Berg-Balance Scale
CF: Center frequency
COM: Center of mass
COP: Center of pressure
CRT: Chair Rising Test
CTSIB: Clinical Test for Sensory Interaction in Balance
Dpas: Passive damping factor
FFb-H-R: Hannover questionnaire for functional disability caused by back pain
Kd: Derivative gain (damping factor)
Ki: Integral gain of the Neural Controller
Kp: Proportional gain (stiffness factor)
LBP: Low back pain
LSS: Lumbar spinal stenosis
MV: Mean velocity
PC-FT: Principal component (PC) of specific functional tests (FT)
POMA: Performance Oriented Mobility Assessment
Ppas: Passive stiffness factor
PROs: Patient-reported outcomes
RMS: Root mean square
SF-36: Short form (36) health survey
Td: Feedback time delay
TUG: Timed Up and Go
Wp: Proprioceptive sensory weight
